# Intraoperative chylous ascites during interval laparoscopic cholecystectomy following acute biliary pancreatitis: a case report from Nepal

**DOI:** 10.1097/RC9.0000000000001178

**Published:** 2026-10-19

**Authors:** Narendra Pandit, Shakil Alam, Bishal Agrawal, Bikash Mandal, Durga Neupane

**Affiliations:** a Department of Surgical Gastroenterology, Birat Medical College and Teaching Hospital, Biratnagar, Nepal; b Department of Surgery, BP Koirala Institute of Health Sciences, Dharan, Nepal

**Keywords:** case report, chylous ascites, conservative management, laparoscopic cholecystectomy, milky-white fluid, pancreatitis, triglycerides

## Abstract

**Introduction:**

Chylous ascites (CA) is an uncommon condition characterized by the accumulation of triglyceride-rich lymphatic fluid within the peritoneal cavity. Acute pancreatitis is an infrequently reported association, and clinically occult CA may be identified unexpectedly during subsequent abdominal surgery.

**Case presentation:**

We report the case of a 72-year-old man who was managed conservatively for moderately severe acute biliary pancreatitis and underwent an interval laparoscopic cholecystectomy 4 weeks later. The initial episode of pancreatitis was characterized by transient organ dysfunction associated with exacerbation of his pre-existing bronchial asthma and a local peripancreatic fluid collection, without persistent organ failure. During surgery, a moderate amount of milky-white peritoneal fluid was incidentally identified. Biochemical analysis of the aspirated fluid showed a triglyceride concentration of 976 mg/dL, supporting the diagnosis of CA. The patient was treated conservatively with a low-fat, medium-chain-triglyceride-rich diet and achieved clinical resolution on short-term follow-up.

**Discussion:**

CA occurring after acute biliary pancreatitis is uncommon and may remain clinically occult. This case highlights the importance of recognizing unexpected intraoperative findings, confirming the diagnosis through ascitic-fluid triglyceride measurement, considering alternative etiologies, and acknowledging that the proposed association with pancreatitis may remain presumptive when direct lymphatic disruption is not demonstrated. Conservative dietary management may be appropriate for selected patients with low-output CA.

**Conclusion:**

Clinically occult CA may persist following apparent recovery from acute biliary pancreatitis and may be discovered incidentally during subsequent abdominal surgery. Early recognition, biochemical confirmation, evaluation for alternative etiologies, and appropriate conservative management may result in favorable short-term clinical outcomes. A causal relationship with pancreatitis cannot be established with certainty in the absence of direct demonstration of lymphatic disruption.

## Introduction

Chylous ascites (CA) is a rare form of ascites characterized by the accumulation of triglyceride-rich lymphatic fluid within the peritoneal cavity^[^1^]^. It results from disruption or obstruction of the lymphatic system, leading to leakage of chyle into the abdominal cavity. The condition may arise secondary to traumatic injury or rupture of lymphatic channels or from elevated lymphatic pressure caused by obstruction of lymphatic flow^[^1^].^ The etiologies of CA are diverse and include traumatic, congenital, infectious, neoplastic, postoperative, cirrhotic, and cardiogenic causes^[^1^]^.

HIGHLIGHTS

- Incidental chylous ascites (CA) was identified during an interval laparoscopic cholecystectomy.
- Acute biliary pancreatitis is an exceptionally rare cause of CA.
- An ascitic fluid triglyceride level of 976 mg/dL confirmed the diagnosis.
- Clinically silent CA persisted despite apparent recovery from pancreatitis.
- A low-fat medium-chain triglyceride diet achieved complete clinical resolution.
- Early recognition and conservative management can avoid unnecessary intervention.

The reported incidence of CA is approximately one in 20 000 hospital admissions, based on data from a large university-based center over a 20-year period^[^2^]^. However, the incidence appears to be increasing, likely due to improved survival among patients with malignancies and to the growing use of complex cardiothoracic and abdominal surgical procedures, including laparoscopic surgery and organ transplantation, which may predispose to lymphatic injury or obstruction^[^3^]^.

Both acute and chronic pancreatitis have been reported infrequently as causes of CA^[^1^]^. The proposed mechanisms include direct disruption of lymphatic channels by pancreatic inflammation, compression of lymphatic vessels by pancreatic edema, pseudocysts, or peripancreatic fluid collections, as well as iatrogenic lymphatic injury during surgical, endoscopic, or radiological interventions performed for the management of pancreatitis.

A recent report described incidental intraoperative CA during laparoscopic cholecystectomy for gallstone pancreatitis^[^4^]^. To our knowledge, the present case is the first reported case from Nepal describing clinically occult CA identified during interval laparoscopic cholecystectomy following acute biliary pancreatitis. We therefore describe the clinical presentation, diagnostic findings, conservative management, and limitations of attributing causality to pancreatitis. This paper has been reported in line with the SCARE 2025 criteria^[^5^]^.

## Case presentation

A 72-year-old man was admitted with acute, moderately severe biliary pancreatitis and was managed conservatively in the high-dependency unit with intravenous fluids, analgesia, and close monitoring. The initial episode was characterized by transient organ dysfunction associated with an exacerbation of his pre-existing bronchial asthma, requiring high-dependency unit care for 12 days. Imaging also demonstrated a local peripancreatic fluid collection. There was no persistent organ failure. He had no other known comorbidities and denied a history of smoking or alcohol consumption.

The diagnosis of acute biliary pancreatitis was established based on clinical and radiological findings. The episode was classified as moderately severe acute pancreatitis based on transient organ dysfunction and the presence of a local complication, without persistent organ failure. Contrast-enhanced computed tomography (CT) demonstrated peripancreatic inflammatory changes and peripancreatic fluid collections without evidence of pancreatic necrosis. The local peripancreatic collection subsequently improved and had resolved by approximately 6 weeks after the index episode. The CT findings also demonstrated gallstones and associated gallbladder inflammatory changes, supporting a biliary etiology. The common bile duct was not dilated, and no pancreatic duct dilatation or focal pancreatic lesion was identified.

Contrast-enhanced CT of the abdomen, performed during the index admission, demonstrated a mildly distended gallbladder containing a few hypodense intraluminal foci measuring up to 5.3 mm, suggestive of gallstones, with diffuse gallbladder wall thickening and surrounding pericholecystic collection (Fig. 1). The common bile duct was not dilated. The pancreas showed an irregular outline with preserved homogeneous enhancement and no focal lesion, calcification, or pancreatic duct dilatation. Peripancreatic fat stranding and peripancreatic fluid collections were noted. Additional findings included mild free fluid in the right subcapsular region, bilateral paracolic gutters, and pelvis, along with inflammatory omental thickening and multiple prominent mesenteric lymph nodes. Bilateral Bosniak grade I renal cortical cysts were also identified.

**Figure 1.**
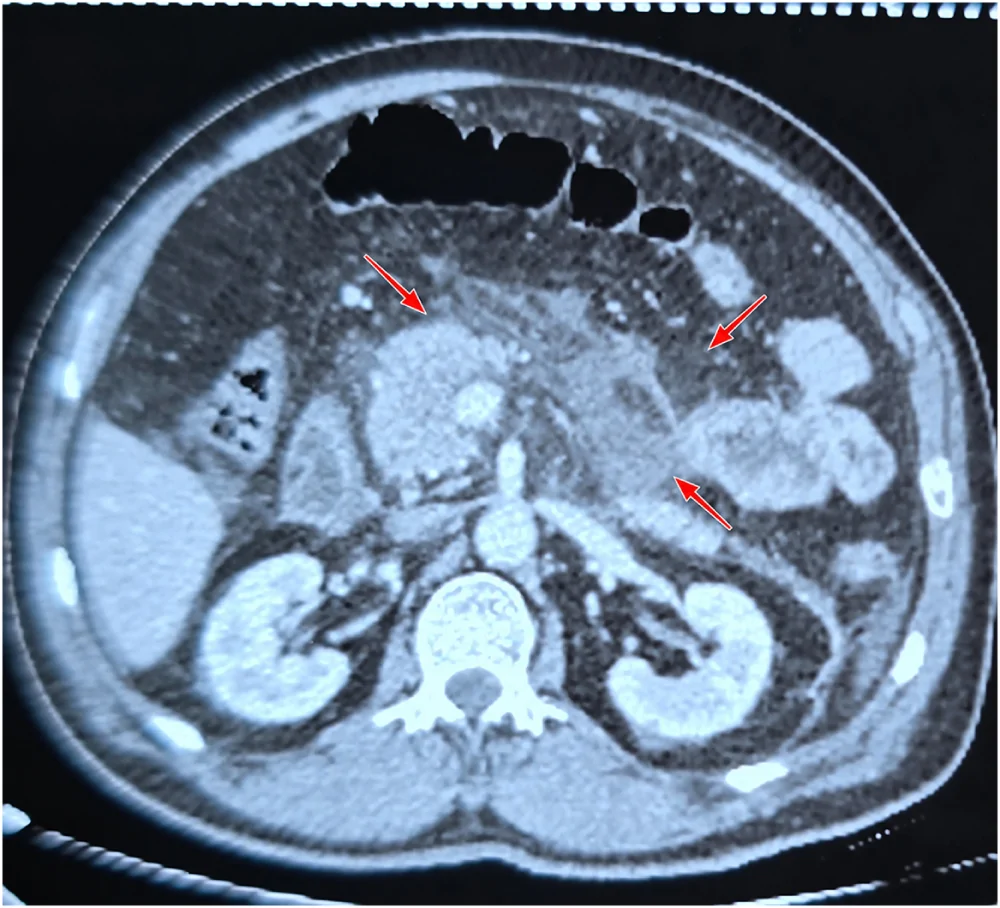
Contrast-enhanced CT of the abdomen during the index episode of acute biliary pancreatitis, demonstrating peripancreatic inflammatory changes, peripancreatic fluid collection, and retroperitoneal inflammatory stranding.

The patient showed clinical improvement and was discharged on day 12 of hospitalization. At a follow-up visit 4 weeks later, he remained asymptomatic, reporting no abdominal pain, fever, nausea, or vomiting, and was tolerating a regular diet. Physical examination revealed stable vital signs and a soft, non-tender abdomen without guarding or rigidity. Follow-up ultrasonography demonstrated multiple gallstones with a normal common bile duct and pancreas. No free intraperitoneal fluid was detected. Laboratory investigations, including complete blood count, liver function tests, renal function tests, and lipid profile, were within normal limits. Therefore, an interval laparoscopic cholecystectomy was planned.

Laparoscopic cholecystectomy was performed under general anesthesia and proceeded uneventfully. However, upon inspection of the abdominal cavity, a moderate amount of milky-white fluid was unexpectedly observed in the pelvis, perisplenic region, and bilateral paracolic gutters (Fig. 2). The liver appeared normal, and there was no evidence of omental saponification, peritoneal deposits, or malignancy. These intraoperative findings raised suspicion for CA.

**Figure 2.**
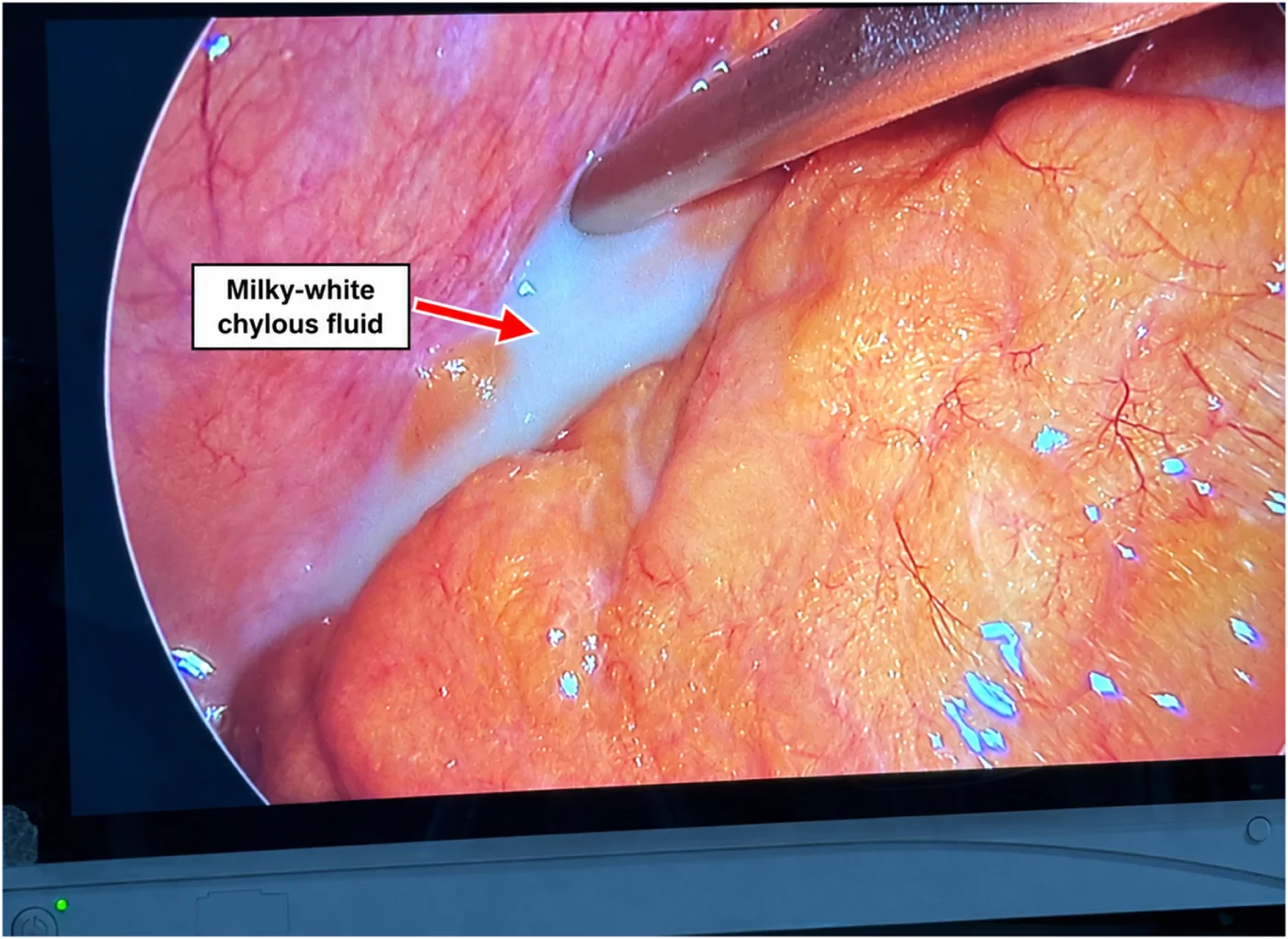
Intraoperative laparoscopic image obtained during an interval cholecystectomy, showing characteristic milky-white chylous fluid within the peritoneal cavity.

The fluid was aspirated and sent for biochemical, microbiological, and cytological analysis. Fluid analysis revealed a triglyceride level of 976 mg/dL, supporting the diagnosis of CA. The fluid culture was sterile. Total leukocyte count was 1170 cells/mm^3^ with lymphocyte predominance (80%); protein concentration was 1.1 g/dL, and glucose level was 34 mg/dL. A pelvic drain was placed at the conclusion of the procedure, yielding approximately 50 mL of chylous fluid daily. Serum-ascitic albumin measurements and ascitic-fluid amylase and other paired biochemical measurements were not available because the chylous fluid was discovered unexpectedly intraoperatively. This limitation is acknowledged in the discussion.

The patient was managed conservatively with a low-fat diet supplemented with medium-chain triglycerides (MCTs). Given the low-output nature of the CA, enteral nutrition was continued. The drain output remained at approximately 50 mL/day without evidence of increased ascites, and the drain was removed on postoperative day 3. The patient remained clinically well, tolerated oral intake, and experienced no abdominal distension. He was discharged on postoperative day 7. Histopathological examination of the gallbladder confirmed chronic calculous cholecystitis. At the 1-month follow-up, the patient remained asymptomatic, maintained his weight, and had no clinical evidence of recurrent ascites. Long-term follow-up was not available and is acknowledged as a limitation.

## Discussion

Abdominal paracentesis is the cornerstone for the diagnosis of CA^[^1^]^. The aspirated fluid typically appears milky, cloudy, or turbid because of its high triglyceride content. Ascitic fluid analysis should include gross examination, cell count and differential, Gram stain, microbiological culture, and measurement of glucose, total protein, albumin, amylase, lactate dehydrogenase, and triglyceride levels^[^6^]^. Cytological examination should also be performed to evaluate for underlying malignancy when clinically indicated. A triglyceride concentration greater than 200 mg/dL in ascitic fluid is generally considered diagnostic of CA, although lower cutoff values have also been proposed in the literature^[^7^]^.

In our patient, CA ascites was discovered incidentally during interval laparoscopic cholecystectomy 4 weeks after acute biliary pancreatitis. The patient was asymptomatic, and preoperative ultrasonography did not demonstrate free intraperitoneal fluid. Intraoperatively, milky-white fluid was identified within the pelvis, perisplenic region, and bilateral paracolic gutters, with a triglyceride concentration of 976 mg/dL, supporting the diagnosis of CA. Several alternative etiologies were considered. There was no history of previous abdominal surgery or trauma, and imaging and intraoperative examination showed no obvious intra-abdominal malignancy or peritoneal deposits. There was no known cirrhosis or chronic liver disease, and the patient had no clinical or radiological evidence of any other obstructive process. Microbiological analysis of the ascitic fluid was sterile, with no clinical evidence suggestive of intra-abdominal infection. In the absence of another identifiable cause, the CA was considered possibly related to lymphatic disruption or obstruction associated with the recent episode of acute biliary pancreatitis. However, a direct lymphatic leak was not demonstrated, and complete paired serum-ascites biochemical characterization was not available; therefore, this causal relationship remains presumptive.

CT and magnetic resonance imaging, although not diagnostic of CA, are valuable imaging modalities for identifying the underlying etiology^[^8^]^. They can help detect intra-abdominal masses, pancreatic or peripancreatic collections, lymphadenopathy, lymphatic obstruction, and other structural abnormalities that may contribute to the development of CA. In the present case, CT imaging during the index episode demonstrated peripancreatic inflammation and fluid collections secondary to acute biliary pancreatitis. These findings provide a plausible mechanism for temporary inflammatory disruption or obstruction of abdominal lymphatic channels, but they do not demonstrate a direct lymphatic leak.

Persistent loss of chyle into the peritoneal cavity can result in significant metabolic, nutritional, and immunological consequences due to the depletion of proteins, lipids, fat-soluble vitamins, electrolytes, immunoglobulins, and fluid^[^1^]^. Although repeated therapeutic paracentesis may provide symptomatic relief, it does not address the underlying lymphatic leak and may exacerbate nutritional deficiencies if definitive treatment is not instituted^[^1^]^. Consequently, nutritional support, including adequate fluid replacement, correction of electrolyte imbalances, and supplementation of nutritional deficiencies, is a key component of management.

Furthermore, the continuous loss of lymphocyte-rich fluid may lead to lymphopenia, hypoproteinemia, and hypogammaglobulinemia, thereby increasing susceptibility to infections^[^1^]^. These potential complications highlight the importance of early diagnosis and timely management of CA to minimize morbidity and improve clinical outcomes.

There is no universally accepted treatment strategy for CA, and management is largely guided by the underlying etiology and the severity of lymphatic leakage. The primary objective is to treat the underlying cause while reducing chyle production, correcting nutritional deficiencies, and preventing metabolic and immunological complications. Conservative management remains the first-line approach in most patients, particularly when the condition is secondary to inflammatory processes and the chyle leak is of low output^[^1^]^.

Dietary modification plays a central role in treatment^[^1^]^. A high-protein, low-fat diet supplemented with MCTs is commonly recommended because MCTs are absorbed directly into the portal venous circulation, thereby reducing intestinal lymphatic flow and chyle production. In patients who fail conservative dietary measures or have high-output chyle leaks, bowel rest, total parenteral nutrition, and pharmacologic agents such as somatostatin or octreotide may be considered^[^9^]^. Surgical or interventional radiologic procedures are generally reserved for refractory cases^[^10^]^.

In the present case, the patient had incidental low-output CA that was identified during an interval laparoscopic cholecystectomy following clinical recovery from acute biliary pancreatitis. No evidence of malignancy, infection, cirrhosis, prior abdominal surgery, or traumatic lymphatic injury was identified. Given the low daily drain output (approximately 50 mL/day) and the patient’s stable clinical condition, conservative management with a low-fat, MCT-rich diet was instituted. The patient remained asymptomatic, tolerated oral intake well, and showed no clinical evidence of recurrent ascites during the 1-month follow-up. However, the short follow-up limits conclusions regarding long-term recurrence.

Acute pancreatitis may contribute to CA through inflammatory disruption or compression of abdominal lymphatic channels caused by pancreatic edema and peripancreatic fluid collections. In the present case, the inflammatory changes and peripancreatic collections observed during the index episode provide a plausible mechanism for temporary lymphatic disruption or obstruction. The absence of pancreatic necrosis or any other identifiable structural cause further supports an inflammatory mechanism; however, the precise lymphatic site of leakage could not be demonstrated. Accordingly, pancreatitis should be regarded as a plausible contributing factor rather than a proven cause.

A 2025 case report by Donaldson and Sutherland described CA discovered intraoperatively during laparoscopic cholecystectomy for gallstone pancreatitis^[^4^]^. Their 58-year-old patient underwent cholecystectomy on day 4 of admission, when milky/purulent fluid was found in all four quadrants; fluid triglycerides were markedly elevated, and cytology showed no malignant cells. The patient was managed with a low-fat, high-protein diet, and the drain was removed 3 weeks later, after cessation of chylous output^[^4^]^. Our case shares the important feature of incidental intraoperative recognition of CA during laparoscopic cholecystectomy in the setting of biliary pancreatitis, but differs in the timing: CA in our patient was clinically occult and identified during interval surgery 4 weeks after the acute episode. The present patient also had documented peripancreatic fluid collections and transient organ dysfunction during the index pancreatitis episode. These similarities and differences suggest that clinically occult CA may persist after apparent clinical improvement, while also emphasizing that a pancreatitis-related mechanism remains presumptive when direct lymphatic disruption is not demonstrated.

This case has several limitations. First, although clinical, radiological, microbiological, and cytological evaluation was used to assess alternative causes where available, a direct lymphatic leak was not demonstrated; therefore, the association between acute biliary pancreatitis and CA remains presumptive. Second, a complete paired serum and ascitic-fluid biochemical evaluation was not available because the CA was discovered unexpectedly during surgery. Third, although the patient received a low-fat, MCT-rich diet and maintained his weight, detailed longitudinal nutritional and biochemical monitoring was limited. Fourth, the duration of clinical follow-up was limited to 1 month, so long-term recurrence cannot be excluded. Nevertheless, the markedly elevated ascitic-fluid triglyceride concentration, temporal relationship with acute biliary pancreatitis, absence of another identifiable etiology, and favorable short-term response to conservative management support the proposed association.

## Conclusions

CA occurring after acute biliary pancreatitis is an uncommon clinical observation. This case demonstrates that clinically occult CA may persist after apparent recovery from pancreatitis and may be discovered incidentally during subsequent abdominal surgery. Prompt recognition, biochemical confirmation, evaluation for alternative etiologies, and appropriate conservative management may result in favorable short-term clinical outcomes. Although a causal relationship cannot be established with certainty in the absence of direct demonstration of lymphatic disruption, acute biliary pancreatitis may represent a plausible contributing factor in selected patients with otherwise unexplained CA.

## Data Availability

All data underlying the results are available as part of the article and no additional source data are required.
